# Engineering mouse cationic trypsinogen for rapid and selective activation by cathepsin B

**DOI:** 10.1038/s41598-019-45631-z

**Published:** 2019-06-24

**Authors:** Alexandra Demcsák, Andrea Geisz, Miklós Sahin-Tóth

**Affiliations:** 1 Center for Exocrine Disorders, Department of Molecular and Cell Biology, Boston University, Henry M. Goldman School of Dental Medicine, Boston, Massachusetts 02118 USA; 2 Department of Surgery, University of California Los Angeles, Los Angeles, California 90095 USA

**Keywords:** Acute pancreatitis, Chronic pancreatitis

## Abstract

Intra-pancreatic activation of trypsin is an early event in pancreatitis. Trypsinogen can be activated to trypsin either through autoactivation (trypsin-mediated trypsinogen activation) or by the lysosomal protease cathepsin B (CTSB). Experimental separation of CTSB-mediated activation from autoactivation in mice is possible through knocking in mutations that render trypsinogen sensitive to CTSB but resistant to trypsin. Here we present biochemical studies on novel mouse cationic trypsinogen (isoform T7) mutants engineered for selective CTSB activation. First, we demonstrated that mutation K24G, which alters the activation site Lys in T7 trypsinogen, abolished autoactivation while activation by CTSB was stimulated 4-fold at pH 4.0. Interestingly, CTSB-mediated activation of the K24G mutant became more sensitive to inhibition by increasing pH. Next, Ala-scanning of the five Asp residues preceding the activation site Lys revealed that mutation D22A accelerated CTSB-mediated activation by 2-fold. Finally, combination of mutations D22A and K24G resulted in a trypsinogen mutant that exhibited 14-fold increased activation by CTSB and normal pH sensitivity. We conclude that we successfully engineered a mouse T7 trypsinogen mutant (D22A,K24G), which is robustly activated by CTSB but cannot undergo autoactivation. These studies set the stage for the generation of a preclinical mouse model of CTSB-dependent pancreatitis.

## Introduction

Activation of the digestive protease trypsinogen to trypsin inside the pancreatic acinar cells is an early event in experimental pancreatitis^1^. The phenomenon has been extensively characterized in rodent models where pancreatitis was induced with supramaximal stimulatory doses of the secretagogue cholecystokinin or its analog cerulein^1–4^ [and references therein]. The exact role of intra-acinar trypsin in disease initiation, however, has remained somewhat of a mystery so far. Genetic manipulations (T7-knockout, *Ctsb*-knockout) that abolished cerulein-induced trypsin activation had only modest protective effects against pancreatitis^5–7^. Conversely, genetically altered mice (*Ctrb1-del*) that exhibited increased cerulein-induced trypsin activation in their pancreas developed more severe pancreatitis^8^. Furthermore, transgenic and knock-in mutant mice (PACE-tryp(on), *T7D23A*) designed to exhibit increased intra-acinar trypsin activation developed spontaneous, progressive pancreatitis^9,10^. Taken together, the observations from animal experiments suggest that trypsin plays a significant role in pancreatitis development and severity only under conditions when intra-pancreatic trypsin activation is stimulated due to genetic alterations. This conclusion agrees well with human genetic studies demonstrating that mutations in susceptibility genes that result in accelerated trypsinogen activation are strong risk factors for recurrent acute and chronic pancreatitis^11^.

The mechanism by which trypsinogen inside acinar cells becomes activated has remained a matter of debate. Activation of trypsinogen to trypsin occurs through limited proteolysis of the Lys-Ile activation-site peptide-bond at the C-terminal end of the trypsinogen activation peptide. Trypsinogen physiologically secreted to the duodenum is activated by enteropeptidase, while inside the pancreas trypsinogen may undergo autoactivation, a self-amplifying bimolecular reaction in which trypsin activates trypsinogen^12^. Mutations in human cationic trypsinogen that stimulate autoactivation cause hereditary pancreatitis;^11,13^ and the disease-causing effect of robust trypsinogen autoactivation was recently recapitulated in a mouse model carrying the D23A mutation in the endogenous mouse cationic trypsinogen (isoform T7)^10^. In rodent models of experimental pancreatitis, however, the lysosomal cysteine protease cathepsin B (CTSB) was identified as a likely activator of trypsinogen^1–4^ [and references therein]. Indeed, CTSB is capable of activating trypsinogen in the test tube^14–21^ and it co-localizes with trypsinogen in intracellular vesicles during cerulein-induced pancreatitis^3,22^. Administration of CTSB inhibitors prevented trypsinogen activation and protected against cerulein-induced pancreatitis^23–25^, although some reports showed no effect^26,27^ and inhibitor specificity remains difficult to ascertain. More convincingly, secretagogue-induced trypsinogen activation was abolished in CTSB-deficient mice, but pancreatitis responses were only modestly mitigated^7^. Human genetic studies provided no compelling evidence for CTSB being a susceptibility gene for pancreatitis. Association with chronic pancreatitis was detected in the Indian population but the effect size was small and could not be replicated in other cohorts^28,29^. *In vitro*, the majority of hereditary-pancreatitis associated cationic trypsinogen mutations that increased autoactivation had no effect or even diminished CTSB-mediated activation^17,18,20^. Thus, a knowledge gap remains whether CTSB-mediated trypsinogen activation observed in rodent models is relevant to the human condition. To address this problem, we set out to generate mouse models in which the two trypsinogen activation processes (autoactivation versus CTSB-mediated activation) can be experimentally separated and their effect on pancreatitis studied selectively. Our autoactivation-dependent spontaneous pancreatitis mouse model (*T7D23A*) was published recently^10^. As a first step towards a CTSB-dependent mouse pancreatitis model, here we performed biochemical studies to identify T7 trypsinogen mutants that cannot autoactivate but are selectively and robustly activated by CTSB.

## Materials and Methods

### Materials

Native human CTSB (catalog number 219362-50UG, 0.427 mg/mL, 233 U/mg protein) and cathepsin L (CTSL) (catalog number 219402-25UG, 0.336 mg/mL, 0.974 U/mg protein) purified from liver were purchased from EMD Millipore (Temecula, California, USA). Before use, the cysteine proteases were incubated with 0.5 mM dithiothreitol (DTT) for 30 min, to fully reduce the active site cysteine. For the experiments presented here, we used CTSB lots 2976367 and 3030153 and CTSL lots 2746070 and 3011670. We observed some differences between the CTSB lots with respect to trypsinogen activation. While initial activation rates were similar, the final trypsin activities slightly differed, suggesting variability in CTSB inactivation during the incubation period. To eliminate this confounding factor, all trypsinogen mutants were purified and tested together with a fresh wild-type T7 trypsinogen preparation and activation curves were compared only within the experiments.

Recombinant mouse CTSB (catalog number 965-CY, 0.44 mg/mL, lot number FOD0318121) was purchased from R&D Systems (Minneapolis, Minnesota, USA). This preparation contains a mixture of proCTSB and mature CTSB. To achieve full activation of the pro form before use, CTSB (1 µL diluted to 5 µL final volume) was incubated in 0.2 M sodium acetate (pH 4.0) and 0.5 mM DTT at 37 °C for 1 hour.

### Nomenclature

Numbering of amino-acid residues in mouse cationic trypsinogen (isoform T7) starts with the initiator methionine of the primary translation product. The first amino acid of mature T7 trypsinogen is Leu16. Compared to human trypsinogens, numbering is shifted by one due to an extra Asp residue in the T7 activation peptide^30^.

### Plasmid construction and mutagenesis

Construction of the bacterial expression plasmid pTrapT7-intein-mouse-T7 harboring the coding sequence for mouse T7 trypsinogen fused to an N-terminal mini-intein was reported previously^10^. Mutations were introduced by overlap-extension PCR mutagenesis.

### Expression and purification of recombinant mouse T7 trypsinogen

Wild-type and mutant T7 trypsinogens were expressed as intein fusion constructs in aminopeptidase P-deficient LG3 *E. coli*. The fusion undergoes spontaneous self-splicing in the cytoplasm, and trypsinogen with an authentic, homogenous N terminus accumulates in inclusion bodies^31^. The isolation of inclusion bodies, *in vitro* refolding and protein purification by ecotin affinity-chromatography were carried out as described previously^32^. Trypsinogen mutants defective in autoactivation were eluted in 50 mM HCl, while mutants prone to autoactivation were eluted in 50 mM HCl containing 100 mM NaCl. Elution conditions for wild-type T7 trypsinogen were matched to those of the mutants purified within the same experiment. The concentration of purified trypsinogen solutions was calculated from their ultraviolet absorbance at 280 nm using the extinction coefficient 39,140 M^−1^cm^−1^.

### Trypsinogen activation with CTSB

Wild-type and mutant forms of T7 trypsinogen were activated at 2 µM concentration with 8.5 µg/mL CTSB (~300 nM) at 37 °C in 0.1 M sodium acetate buffer (pH 4.0, 4.5 or 5.0, as indicated), 1 mM K-EDTA and 0.05% Tween 20, in 100 µL final volume. The reaction was initiated by adding 2 µL CTSB and at the indicated times 2 µL aliquots were withdrawn and mixed with 48 µL assay buffer (0.1 M Tris-HCl (pH 8.0), 1 mM CaCl_2_ and 0.05% Tween 20). Trypsin activity was measured by adding 150 µL of 200 µM *N*-CBZ-Gly-Pro-Arg-*p*-nitroanilide substrate (dissolved in assay buffer) and following the release of the yellow p-nitroaniline at 405 nm in a microplate reader. Rates of substrate cleavage were determined from the initial linear portions of the curves and expressed in mOD/min units. For gel electrophoresis, incubations were carried out at pH 4.0 in the absence of Tween 20 and the reactions were analyzed as described below.

The activation of wild-type T7 trypsinogen and the D22A,K24G mutant was also tested with recombinant mouse CTSB. In this case, the reaction was initiated by adding 1 µL of mouse CTSB pretreated as described under *Materials*.

### Trypsinogen inactivation with CTSL

Wild-type and mutant forms of T7 trypsinogen were digested at 2 µM concentration with 8.4 µg/mL CTSL (~290 nM) at 37 °C in 0.1 M sodium acetate buffer (pH 4.0), 1 mM K-EDTA and 0.05% Tween 20, in 100 µL final volume. The reaction was initiated by adding 2.5 µL CTSL and at the indicated times 2 µL aliquots were withdrawn and trypsin activity was measured as described above. For gel electrophoresis, incubations were carried out in the absence of Tween 20 and the reactions were analyzed as described below.

### SDS-PAGE analysis

At the indicated times, the CTSB and CTSL reaction mixtures (100 µL) were precipitated with 10% trichloroacetic acid (final concentration); the precipitate was pelleted by centrifugation and dissolved in 20 µL Laemmli sample buffer containing 100 mM DTT. Samples were heat-denatured at 95 °C for 5 min, followed by electrophoresis on 15% SDS-polyacrylamide minigels. Bands were visualized by staining the gels with Coomassie Blue R-250.

## Results

### Mutation K24G in trypsinogen imparts CTSB selectivity

**Figure 1:**
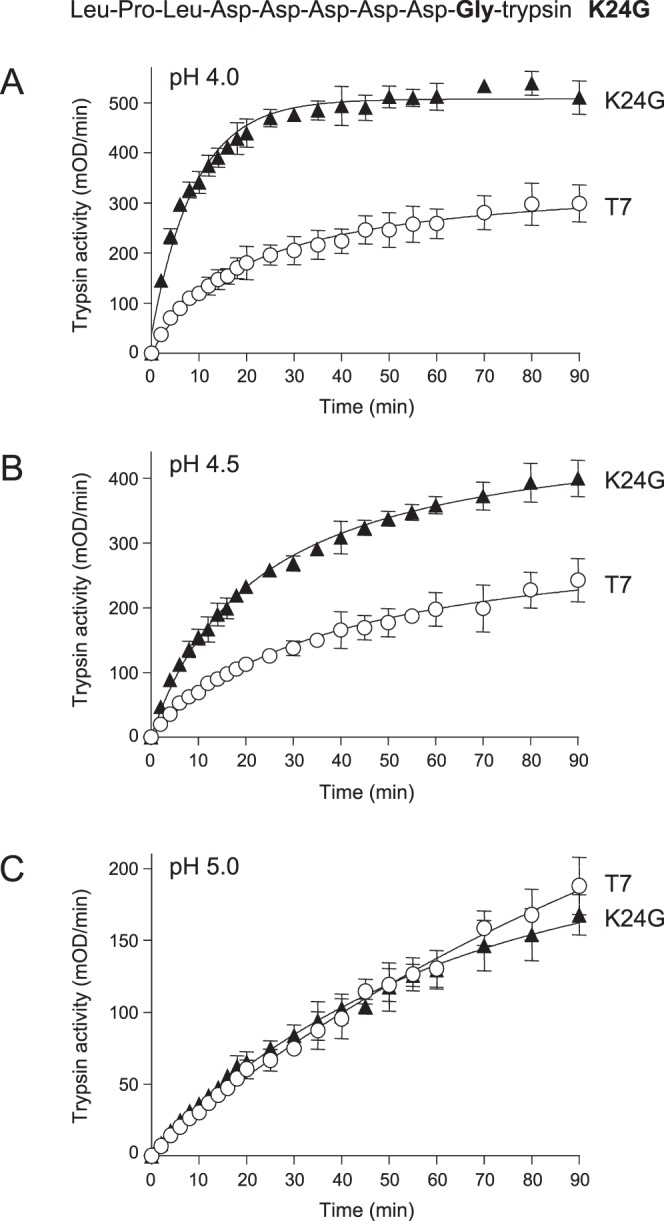
Activation of wild-type (T7) mouse cationic trypsinogen and mutant K24G with human cathepsin B (CTSB). Trypsinogens were incubated with CTSB at (**A**), pH 4.0, (**B**), pH 4.5 and (**C**), pH 5.0, and trypsin activity was measured, as described in *Methods*. Mean values with S.D. (n = 3) are shown. The amino-acid sequence of the T7 activation peptide with mutation K24G is also indicated.

To prevent trypsinogen activation by trypsin (autoactivation) but still maintain CTSB-mediated activation, we mutated the Lys residue to Gly (mutation K24G) in the Lys24-Ile25 activation-site peptide-bond of T7 trypsinogen (Fig. 1). We chose Gly as the replacement because studies on CTSB substrate specificity demonstrated that a P1 Gly is a preferred residue for efficient proteolysis^33,34^. P1 designates the N-terminal amino-acid residue in a cleaved peptide bond. Aromatic and aliphatic P1 residues were excluded from consideration because pancreatic chymotrypsins and elastases might cleave after these and cause unwanted trypsinogen activation. As expected, mutant K24G was completely deficient in autoactivation (not shown). Next, we measured activation of wild-type and K24G mutant T7 trypsinogen by human CTSB at pH 4.0, 4.5 and 5.0. Remarkably, mutant K24G was activated 4-fold faster by CTSB than wild-type T7 trypsinogen at pH 4.0 (Fig. 1A, Table 1). As shown before^18^, increasing the pH from 4.0 to 5.0 inhibited the activation reaction; the rate for wild-type T7 decreased by about 6-fold. Interestingly, activation of mutant K24G was more sensitive to pH inhibition and its activation rate at pH 5.0 was reduced by almost 19-fold relative to pH 4.0 (Fig. 1B,C, Table 1). Consequently, the difference in the activation rates between the K24G mutant and wild-type T7 diminished with increasing pH and at pH 5.0 the two activation reactions were essentially superimposable (Fig. 1C). Finally, we performed control experiments with porcine elastase 1 to exclude the possibility of elastase-mediated activation, as elastases may cleave after Gly residues. Using 200 nM elastase as activator at pH 8.0, we observed no activation of the K24G trypsinogen mutant (not shown).

**Table 1:** Initial rates of cathepsin B-mediated trypsinogen activation reactions.

pH	Trypsinogen	Rate	Relative rate
4.0	K24G	72.5	4.1
	T7	17.7	
4.5	K24G	23.6	2.6
	T7	9	
5.0	K24G	3.9	1.3
	T7	3	
4.0	D19A	18.5	1
	T7	18	
4.0	D20A	16.1	0.9
	T7	18	
4.0	D21A	16.1	0.7
	T7	21.8	
4.0	D22A	41.6	1.9
	T7	21.8	
4.0	D23A	10.9	0.5
	T7	21.2	
4.0	D22A,K24G	202.1	13.6
	T7	14.9	
4.5	D22A,K24G	123.2	15
	T7	8.2	
5.0	D22A,K24G	45.8	18.3
	T7	2.5	
4.0	D23A,K24G	35.6	1.9
	T7	19.1	
4.5	D23A,K24G	17.9	1.6
	T7	11.4	
5.0	D23A,K24G	4.4	1.1
	T7	4.1	
4.0	D22A,D23A,K24G	123.6	10.3
	T7	12	
4.5	D22A,D23A,K24G	83.7	13.7
	T7	6.1	
5.0	D22A,D23A,K24G	33.9	15.4
	T7	2.2	
**Activation with mouse CTSB**			
4.0	D22A,K24G	87.2	11.2
	T7	7.8	
5.0	D22A,K24G	23.3	9.3
	T7	2.5	

Rates were determined from the initial, quasi-linear portions of the activation curves and expressed in mOD/min/min units. Relative rates for mutants were calculated with respect to the activation rate of wild-type mouse cationic trypsinogen (T7).

### Ala-scanning reveals mutation D22A stimulates CTSB-mediated trypsinogen activation

**Figure 2:**
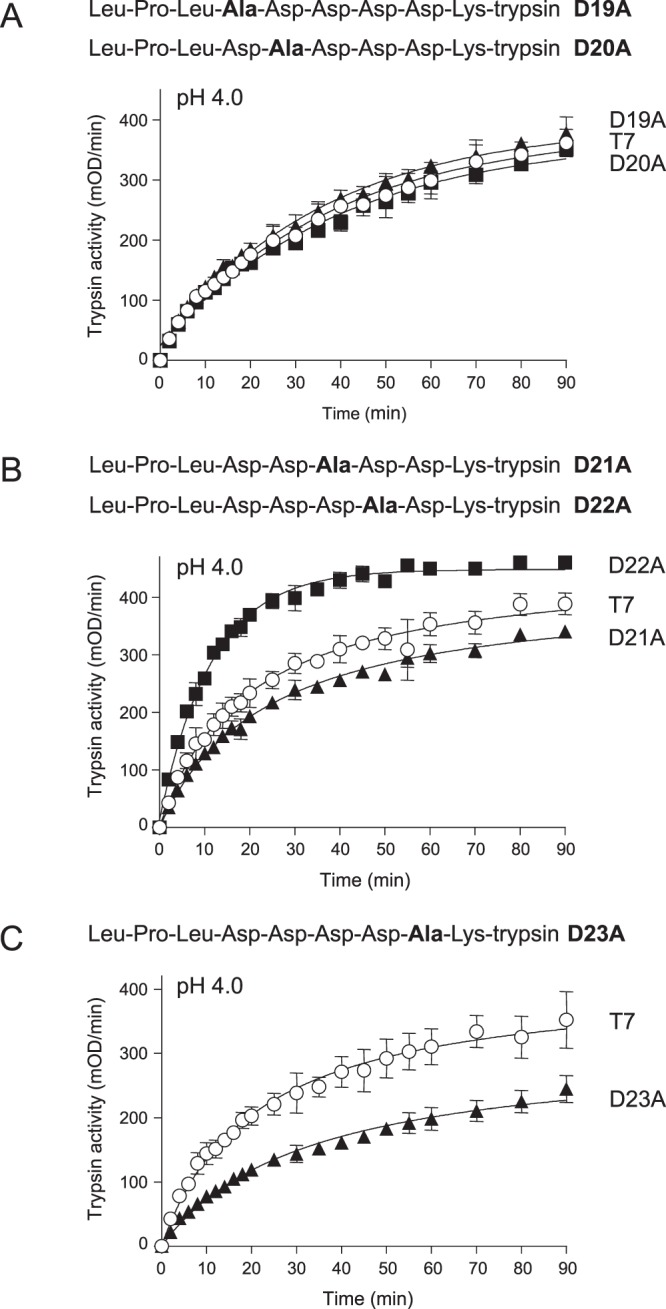
Activation of wild-type (T7) mouse cationic trypsinogen and mutants D19A, D20A, D21A, D22A and D23A with human cathepsin B (CTSB). Trypsinogens were incubated with CTSB at pH 4.0, and trypsin activity was measured, as described in *Methods*. Mean values with S.D. (n = 3) are shown. (**A**), mutants D19A and D20A, (**B**), mutants D21A and D22A, (**C**), mutant D23A. The amino-acid sequences of the T7 activation peptides with the various mutations are also indicated.

The significance of the penta-Asp motif preceding the activation site in the T7 trypsinogen activation peptide was assessed by individually mutating the five Asp residues to Ala and measuring CTSB-mediated activation at pH 4.0. In these constructs, the activation site Lys was intact, therefore, they were prone to autoactivation. To rule out the confounding effect of autoactivation, we employed higher salt concentration (100 mM NaCl) during purification and we performed the CTSB-dependent activation experiment at pH 4.0 only. We found that mutations D19A and D20A had no effect on CTSB-mediated trypsinogen activation (Fig. 2A), while mutation D22A stimulated the reaction about 2-fold (Fig. 2B, Table 1). Finally, mutations D21A and D23A inhibited activation by about 25% and 50%, respectively (Fig. 2B,C, Table 1).

### Activation of double mutant D22A,K24G by CTSB is markedly increased

**Figure 3:**
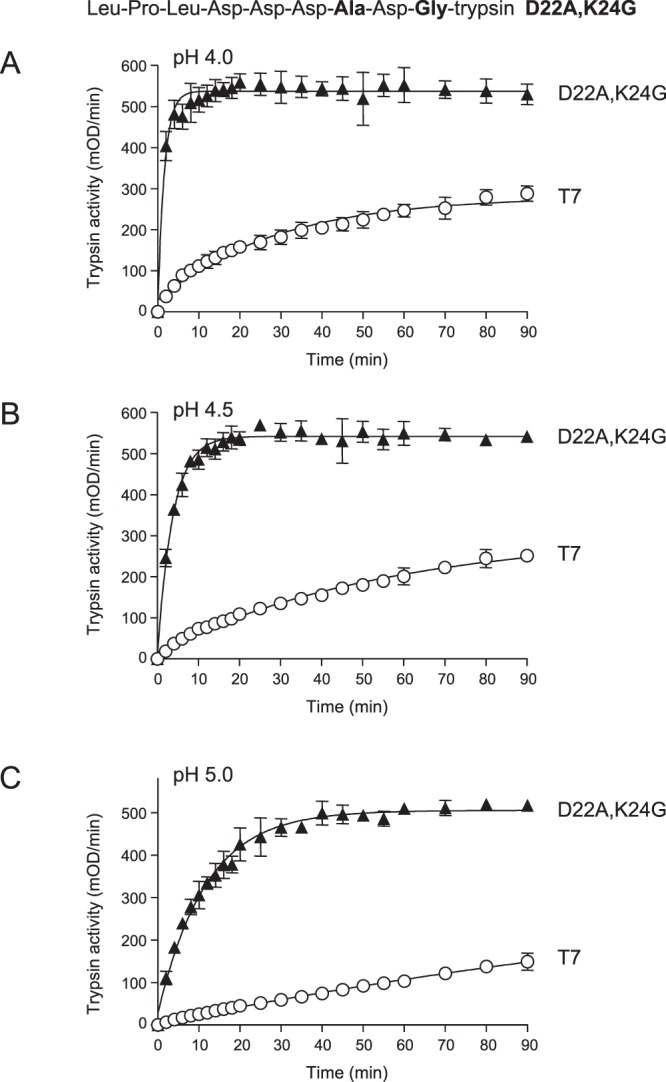
Activation of wild-type (T7) mouse cationic trypsinogen and mutant D22A,K24G with human cathepsin B (CTSB). Trypsinogens were incubated with CTSB at (**A**), pH 4.0, (**B**), pH 4.5 and (**C**), pH 5.0, and trypsin activity was measured, as described in *Methods*. Mean values with S.D. (n = 3) are shown. The amino-acid sequence of the T7 activation peptide with mutations D22A,K24G is also indicated.

Combination of mutations D22A and K24G resulted in a trypsinogen mutant, which was activated by CTSB at 14-fold increased rate at pH 4.0 (Fig. 3A). Importantly, in contrast to the K24G single mutant, activation of double mutant D22A,K24G did not show increased sensitivity to higher pH and the activation rates decreased at pH 4.5 and pH 5.0 in the same manner as seen with wild-type T7 trypsinogen (Fig. 3B,C, Table 1). Consequently, the difference in rates of activation between wild-type T7 and D22A,K24G mutant trypsinogens remained high over the entire pH range from 4.0 to 5.0 (Fig. 3, Table 1). The observations also imply that deprotonation of Asp22 was responsible for the sharp inhibition of the activation of mutant K24G at higher pH (see Fig. 1).

**Figure 4:**
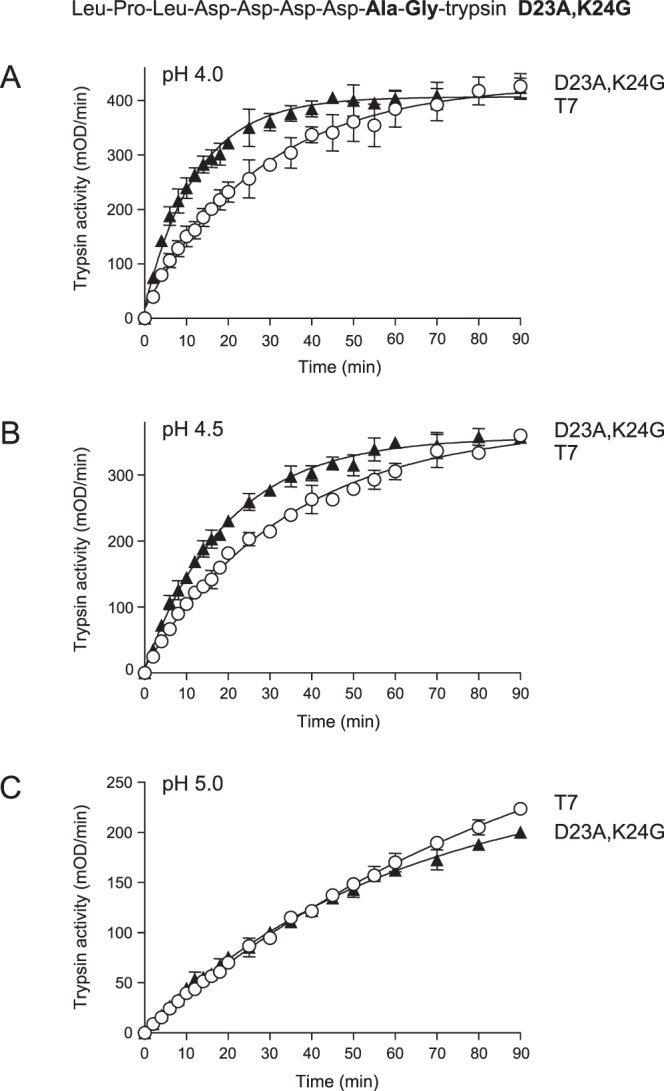
Activation of wild-type (T7) mouse cationic trypsinogen and mutant D23A,K24G with human cathepsin B (CTSB). Trypsinogens were incubated with CTSB at (**A**), pH 4.0, (**B**), pH 4.5 and (**C**), pH 5.0, and trypsin activity was measured, as described in *Methods*. Mean values with S.D. (n = 3) are shown. The amino-acid sequence of the T7 activation peptide with mutations D23A,K24G is also indicated.

As a control experiment, we also prepared double mutant D23A,K24G and tested its activation by CTSB. As shown in Fig. 4, this double mutant was activated at rates that were only slightly higher than those of wild-type T7 trypsinogen at all pH values tested (Table 1). When compared to the single mutant K24G (see Fig. 1), the addition of mutation D23A significantly decreased CTSB-mediated activation at pH 4.0 and pH 4.5.

### Activation of triple mutant D22A,D23A,K24G is not improved over D22A,K24G

**Figure 5:**
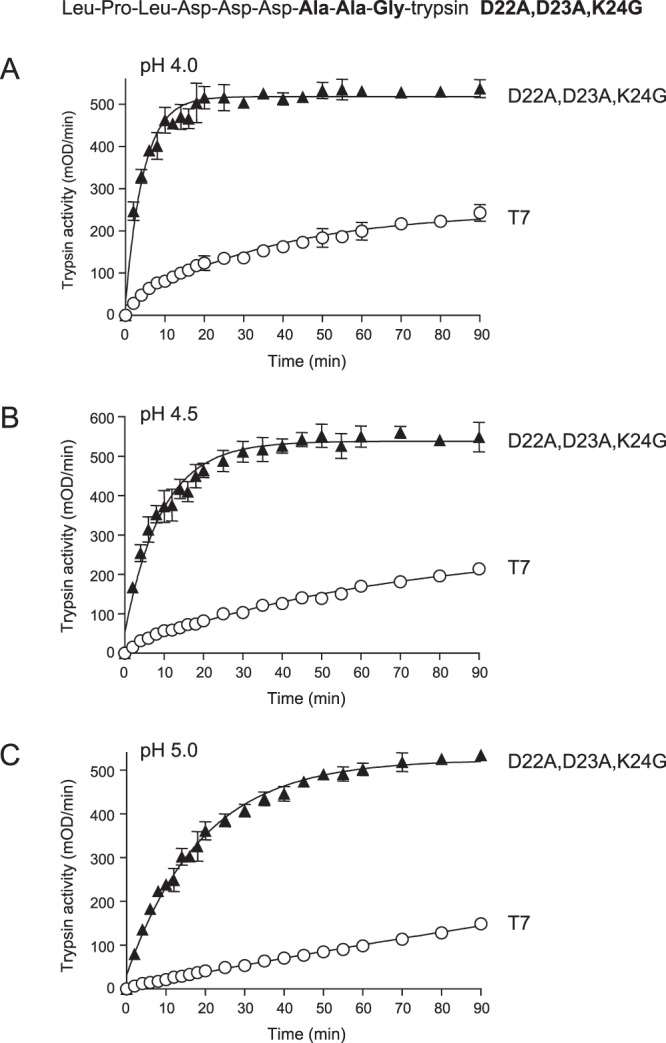
Activation of wild-type (T7) mouse cationic trypsinogen and mutant D22A,D23A,K24G with human cathepsin B (CTSB). Trypsinogens were incubated with CTSB at (**A**), pH 4.0, (**B**), pH 4.5 and (**C**), pH 5.0, and trypsin activity was measured, as described in *Methods*. Mean values with S.D. (n = 3) are shown. The amino-acid sequence of the T7 activation peptide with mutations D22A,D23A,K24G is also indicated.

We also considered the possibility that mutation D23A in the context of double mutant D22A,K24G may further improve CTSB-mediated activation. Although single mutant D23A and double mutant D23A,K24G showed decreased or unchanged activation characteristics relative to wild-type T7 trypsinogen, we still speculated that the D22A,D23A,K24G triple mutant may have a different effect. Indeed, the triple mutant showed robustly enhanced activation by CTSB when compared to wild-type T7 trypsinogen (Fig. 5), however, there was no improvement when the comparison with double mutant D22A,K24G was made; rates of activation were slightly slower (cf. Fig. 3, Table 1).

### SDS-PAGE analysis of the CTSB-mediated activation reactions

**Figure 6:**
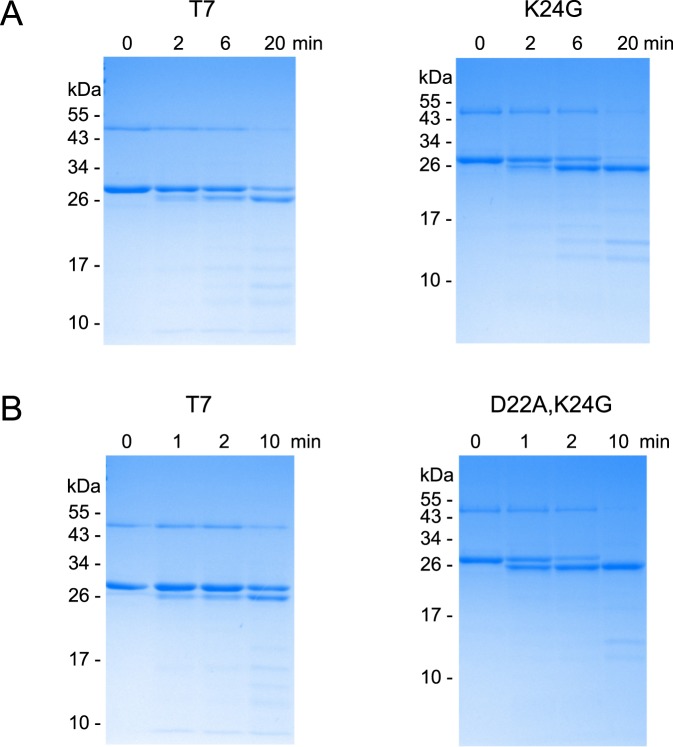
Activation of wild-type (T7) mouse cationic trypsinogen and mutants K24G and D22A,K24G with human cathepsin B (CTSB). Trypsinogens were incubated with CTSB at pH 4.0, as described in *Methods*. At the indicated times, samples were precipitated with trichloroacetic acid and analyzed by sodium dodecyl sulfate-polyacrylamide gel electrophoresis and Coomassie Blue staining, as described in *Methods*. Representative gels from two experiments are shown. (**A**), mutant K24G, (**B**), mutant D22A,K24G. The two major bands above the 26 kDa marker correspond to T7 trypsinogen and trypsin. The faint band above the 43 kDa marker is intein-trypsinogen fusion protein.

To visualize the activation reactions of mutants K24G and D22A,K24G, we performed polyacrylamide gel electrophoresis followed by Commassie Blue staining (Fig. 6). We observed the typical band shift in migration as the trypsinogen band was converted to trypsin over time. In agreement with the activity measurements, both mutants were activated more rapidly than wild-type T7 trypsinogen. Trypsinogen degradation was minimal, as judged by the comparable intensities of the trypsinogen and trypsin bands and the presence of faint lower molecular weight bands.

### Proteolytic inactivation of mutants K24G and D22A,K24G by CTSL

**Figure 7:**
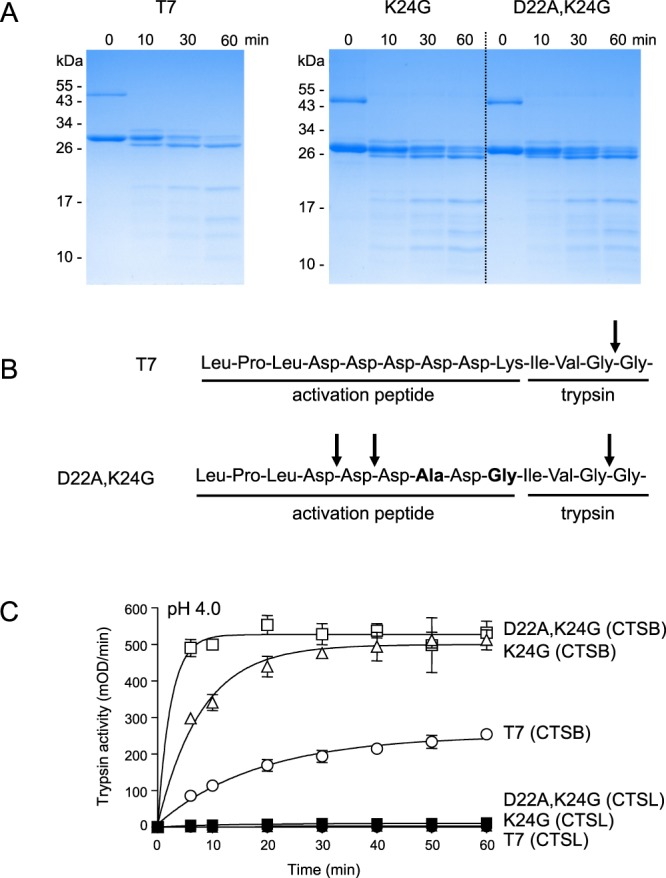
Inactivation of wild-type (T7) mouse cationic trypsinogen and mutants K24G and D22A,K24G by human cathepsin L (CTSL). Trypsinogens were incubated with CTSL at pH 4.0, as described in *Methods*. (**A**), At the indicated times, samples were precipitated with trichloroacetic acid and analyzed by sodium dodecyl sulfate-polyacrylamide gel electrophoresis and Coomassie Blue staining, as described in *Methods*. Representative gels from two experiments are shown. The two major bands above the 26 kDa marker correspond to intact T7 trypsinogen and CTSL-cleaved forms. The faint band above the 43 kDa marker is intein-trypsinogen fusion protein. (**B**), Trypsinogen bands were transferred to a polyvinylidene fluoride (PVDF) membrane and subjected to N-terminal sequence analysis by Edman degradation. The arrows indicate the deduced CTSL cleavage sites within the N-terminal region of trypsinogen. (**C**), Trypsin activity of the CTSL incubates was measured, as described in *Methods*. For comparison, the CTSB activation curves are also shown. Mean values with S.D. (n = 3) were plotted.

In contrast to CTSB, the other major lysosomal cysteine protease CTSL does not activate trypsinogen. Instead, CTSL was shown to inactivate bovine and human trypsinogen and trypsin by cleaving after Gly26, which corresponds to Gly27 in mouse T7 trypsinogen^35^. To examine whether mutations K24G and D22A,K24G alter CTSL-mediated inactivation, we treated the mutants with human CTSL and analyzed the cleavage reaction by SDS-PAGE and Coomassie Blue staining (Fig. 7A). Cleavage sites in wild-type T7 and the D22A,K24G mutant were verified by N-terminal protein sequencing of the digested trypsinogen bands via Edman degradation (Fig. 7B). Wild-type T7 was cleaved at the expected site, i.e. after Gly27 and a single major cleavage product was generated. Mutant D22A,K24G was cleaved slightly slower at the same site and an additional intermediate band was also generated as a consequence of cleavages after Asp19 and Asp20. Finally, consistent with the inactivating cleavages observed, trypsin activity measurements confirmed that CTSL did not activate wild-type T7 or mutants K24G and D22A,K24G to an appreciable extent (Fig. 7C).

### Activation of double mutant D22A,K24G by mouse CTSB

**Figure 8:**
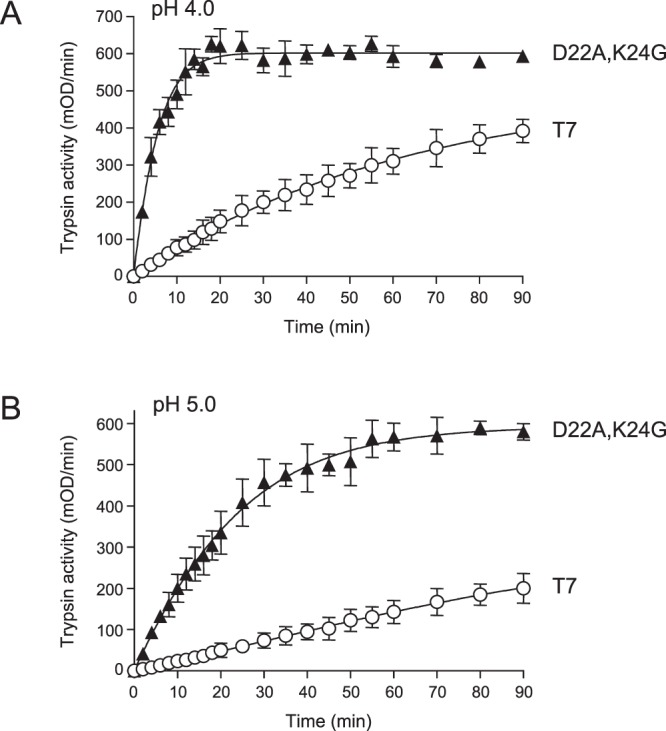
Activation of wild-type (T7) mouse cationic trypsinogen and mutant D22A,K24G with mouse cathepsin B (CTSB). Trypsinogens were incubated with mouse CTSB at (**A**), pH 4.0 and (**B**), pH 5.0, and trypsin activity was measured, as described in *Methods*. Mean values with S.D. (n = 3) are shown.

The studies described above were carried out with a human CTSB preparation purified from liver, which has been commercially available for decades and has been used by many laboratories. However, in our planned mouse model, the D22A,K24G trypsinogen mutant will be activated by native mouse CTSB, the mature form of which shares 84% sequence identity with its human counterpart. To rule out species-specific differences in trypsinogen activation by CTSB, we performed experiments with recombinant mouse CTSB at pH 4.0 and pH 5.0. At both pH values, trypsinogen mutant D22A,K24G was activated about 10-fold more rapidly than wild-type T7 (Fig. 8, Table 1).

## Discussion

In the present study, we engineered the mouse cationic trypsinogen for robust and selective activation by CTSB. This was achieved by strategically mutating the trypsinogen activation peptide so that trypsin-mediated activation (autoactivation) was abolished while CTSB-mediated activation was accelerated. Inhibition of autoactivation was achieved by mutation K24G, which eliminated the activation site Lys and increased CTSB-sensitivity 4-fold at pH 4.0. Interestingly, as the pH was increased from 4.0 to 5.0, the difference between CTSB-mediated activation of T7 trypsinogen and the K24G mutant disappeared. This effect was due to the stronger inhibition of CTSB-mediated activation of mutant K24G by increasing pH. Further enhancement of CTSB-dependent activation of the K24G mutant was accomplished by the introduction of mutation D22A. The D22A,K24G double mutant was activated by CTSB at least 14-fold more rapidly than wild-type T7 trypsinogen and this difference was maintained over the pH range from 4.0 to 5.0. Importantly, activation with mouse CTSB versus human CTSB yielded similar results.

The behavior of mutants K24G and D22A,K24G with respect to inhibition of CTSB-mediated trypsinogen activation at pH 5.0 suggests that the protonation state of Asp22 is an important determinant of the reaction and a deprotonated, negatively charged Asp is inhibitory. The intrinsic pKa of Asp residues in peptide chains is 3.9 and the average pKa in proteins is around 3.5^36^. This suggests that Asp22 in mutant K24G likely carries a partial negative charge at pH 4.0, which becomes even more negative as the pH is increased to 5.0 and Asp22 becomes more fully deprotonated. Mutation D22A neutralizes the charge, stimulates CTSB-mediated activation of mutant K24G and renders it less prone to pH-inhibition. Curiously, wild-type T7 trypsinogen shows similar pH-sensitivity as mutant D22A,K24G, in all likelihood due to an interaction between the positively charged side chain of the activation site Lys24 and one or more of the preceding Asp residues, possibly Asp22.

CTSL co-localizes with CTSB and it can inactivate trypsinogen and trypsin through proteolytic cleavage of the conserved Gly26-Gly27 peptide bond in human and bovine trypsinogens^35^. Mouse T7 trypsinogen is also digested by CTSL at the corresponding Gly27-Gly28 peptide bond, resulting in a truncated, inactive trypsin(ogen) species (see Fig. 7). In contrast to the marked stimulatory effect on CTSB-mediated activation, mutations K24G and D22A,K24G had no significant impact on CTSL-mediated degradation. In fact, the mutants were cleaved slightly slower by CTSL, which is expected to further enhance CTSB-mediated activation in the acinar cell.

The purpose of these biochemical studies was to set the stage for the generation of a knock-in mouse model carrying the D22A,K24G mutations in T7 trypsinogen. Upon co-localization of lysosomes and zymogen granules in the pancreas of these mice, trypsinogen is expected to undergo robust activation, which should result in spontaneous pancreatitis or markedly elevated sensitivity to cerulein-induced pancreatitis. Ideally, a CTSB-dependent preclinical pancreatitis model can be created, which will be useful for testing therapeutic approaches targeting CTSB. Furthermore, the mouse model would be important conceptually, as the exact role for CTSB in pancreatitis cannot be convincingly ascertained by the phenotype of the CTSB-deficient mice^7^. CTSB was globally deleted in these animals and it is unclear what effect that might have had on the inflammatory response. Inside the pancreatic acinar cells, CTSB was shown to participate not only in trypsinogen activation but also in mediating cell death, both necrosis and apoptosis^37,38^ and it may have other cellular effects as well. Thus, CTSB deficiency can affect multiple aspects of the pancreatitis response and the final phenotypic outcome may be mechanistically misleading. A similar situation arose when trypsin activation and pancreatitis responses were studied in CTSL-deficient mice^35^. Even though trypsin activation was markedly increased in these mice, pancreatitis was still ameliorated, in all likelihood due to other effects of CTSL-deletion on immune cells and cell death pathways. In the approach we are proposing, we will leave all other CTSB functions untouched and only manipulate one substrate for CTSB, i.e. trypsinogen. In this way, any disease phenotype observed can be directly related to changes in CTSB-mediated trypsinogen activation.

Experimental separation of autoactivation from CTSB-mediated activation in genetically-modified mice was recently achieved when we created a mouse model carrying the D23A mutation in the T7 trypsinogen activation peptide^10^. The D23A mutation selectively increases autoactivation, while CTSB-mediated activation is slightly inhibited and CTSL-mediated cleavage is unchanged. The *T7D23A* mice develop early onset spontaneous acute pancreatitis followed by progressive chronic pancreatitis with end-stage disease characterized by acinar cell atrophy and adipose replacement. The model offered proof that increased trypsinogen autoactivation can drive pancreatitis onset and progression; a notion that supports similar conclusions derived from human genetic and biochemical studies^11^. Nonetheless, the *T7D23A* model does not rule out the possibility that CTSB-mediated trypsinogen activation can be equally effective in promoting pancreatitis with or without trypsinogen autoactivation. The generation of a mouse model with the D22A,K24G trypsinogen mutant will directly address this question.

In conclusion, we constructed and characterized new mutant forms of mouse cationic trypsinogen (isoform T7), which can be robustly and selectively activated by CTSB but cannot undergo autoactivation. These studies facilitate the design of mouse models that will inform on the role of CTSB-mediated trypsinogen activation in the development of pancreatitis.

## Data Availability

Materials, data and protocols associated with this paper are available upon request.
